# Efficacy of the Geriatric Nutritional Risk Index for Predicting Overall Survival in Patients with Head and Neck Cancer: A Meta-Analysis

**DOI:** 10.3390/nu15204348

**Published:** 2023-10-12

**Authors:** Ching-Yi Yiu, Chien-Cheng Liu, Jheng-Yan Wu, Wen-Wen Tsai, Ping-Hsin Liu, Wan-Jung Cheng, Jen-Yin Chen, Kuo-Chuan Hung

**Affiliations:** 1 Department of Otolaryngology, Chi Mei Medical Center, Liouying, Tainan City 73657, Taiwan; 2 Department of Dental Laboratory Technology, Min-Hwei Junior College of Health Care Management, Liouying, Tainan City 73658, Taiwan; 3 Department of Anesthesiology, E-Da Hospital, I-Shou University, Kaohsiung City 82445, Taiwan; 4 Department of Nursing, College of Medicine, I-Shou University, Kaohsiung City 82445, Taiwan; 5 School of Medicine, I-Shou University, Kaohsiung City 82445, Taiwan; 6 Department of Nutrition, Chi Mei Medical Center, Tainan City 71004, Taiwan; 7 Department of Neurology, Chi Mei Medical Center, Tainan City 71004, Taiwan; 8 Department of Anesthesiology, E-Da Dachang Hospital, I-Shou University, Kaohsiung City 82445, Taiwan; 9 Department of Anesthesiology, Chi Mei Medical Center, Liouying, Tainan City 73657, Taiwan; 10 Department of Anesthesiology, Chi Mei Medical Center, Tainan City 71004, Taiwan; 11 School of Medicine, College of Medicine, National Sun Yat-sen University, Kaohsiung City 804201, Taiwan

**Keywords:** geriatric nutritional risk index, head and neck cancer, overall survival, meta-analysis

## Abstract

Head and neck cancer (HNC) is a prevalent malignancy with a poor prognosis, necessitating the identification of prognostic biomarkers to guide management. The geriatric nutritional risk index (GNRI), calculated from serum albumin and body weight, may predict survival in patients with HNC. We performed a systematic review and meta-analysis to clarify this relationship. Databases were searched for studies examining the association between pretreatment GNRI and overall survival in patients with HNC. Ten studies with 2793 patients were included. Meta-analysis demonstrated that low GNRI was associated with significantly worse overall survival compared to high GNRI (hazard ratio [HR]:2.84, 95% CI 2.07–3.91, *p* < 0.00001). Older age (HR:1.73; 95% CI, 1.35–2.22; *p* < 0.0001), male sex (HR:1.7; 95% CI, 1.12–2.6; *p* = 0.01), advanced tumor stage (HR: 2.5; 95% CI, 1.72–3.63; *p* < 0.00001), and higher T-/*N*-stage (HR = 1.69 and 1.98, respectively) were also predictive of unfavorable outcomes. The GNRI had the highest HR, suggesting potent predictive ability. Despite limitations, including retrospective design and potential publication bias, our study indicates that low pretreatment GNRI predicts poor overall survival in patients with HNC. The GNRI is an inexpensive, routinely available biomarker that could improve prognostication and guide management decisions. Additional research is warranted to validate these findings.

## 1. Introduction

Head and neck cancer (HNC), which frequently originates from the mucosal epithelium of the oral cavity, pharynx, and larynx, is the seventh most prevalent cancer type worldwide, with over 660,000 new diagnoses and approximately 325,000 fatalities annually [1,2]. The incidence of HNC is expected to increase by 30% annually by 2030 [1,2], affecting both developed and developing countries. The major risk factors for HNC include tobacco smoking and combined alcohol consumption; regional influences, such as areca nut chewing, increase oral cancer rates in Southeast Asia and the Asia–Pacific, whereas oropharyngeal cancers linked to human papillomavirus contribute to the increasing incidence of HNC in the United States and Europe [3,4,5,6]. The global five-year survival rate for HNC is approximately 50% [7]. While the mortality rates have remained relatively stable, survival outcomes significantly differ based on geographical location, tumor site, and stage at diagnosis [8]. In the United Kingdom, HNC mortality rates have steadily increased over the past decade, possibly due to the increasing incidence and stagnating survival rates [5]. There are several patient- and tumor-related factors that influence clinical outcomes in HNC. Therefore, the identification of prognostic biomarkers that can improve risk stratification and guide treatment decisions has attracted attention [9,10,11,12].

Cumulative evidence suggests that nutritional status and systemic inflammation play a significant role in cancer progression and survival [13,14,15]. The geriatric nutritional risk index (GNRI), calculated from serum albumin concentration and body weight, is a simple nutritional screening tool that may have a prognostic value in malignancies. It reflects both nutritional status and inflammation, with lower scores indicating a worse prognosis. In the current literature, the GNRI has been examined as a predictor of clinical outcomes in various cancers, including esophageal, gastric, colorectal, and lung cancers [16,17,18,19]. For example, in a meta-analysis of 14 retrospective studies including 3981 patients with esophageal cancer, the aggregated data indicated that a low pretreatment GNRI can serve as an independent prognostic determinant for reduced overall survival, with a hazard ratio (HR) of 1.47 [16]. While some individual studies suggest that the GNRI may predict overall survival in patients with HNC [20,21,22], these findings are limited by small sample sizes, indicating that pooled evidence is needed to definitively establish the role of the GNRI in predicting survival for these patients. We aimed to conduct a systematic review and meta-analysis of the association between pretreatment GNRI and overall survival in patients with HNC. Elucidating the utility of this inexpensive and routinely available biomarker could help better risk-stratify patients and individualize treatment decisions.

## 2. Materials and Methods

### 2.1. Data Source and Protocol Registration

To identify studies examining the association between pretreatment GNRI and overall survival in patients with HNC, four electronic databases, namely, Embase (from 1974 to 1 September 2023), MEDLINE (from 1946 to 1 September 2023), Google Scholar, and Cochrane Central Register of Controlled Trials, were screened. The electronic search was performed on 3 September 2023 without language limitation. The following free-text words were used: “head and neck cancer,” “head and neck neoplasm,” “head and neck tumor,” “oral cancer,” “larynx cancer,” “pharynx cancer,” “geriatric nutritional risk index,” “GNRI,” and “overall survival.” Additionally, medical subject headings were used to facilitate the literature search. A summary of details using MEDLINE as an example is presented in Table 1. To identify further relevant reports, cited references of relevant studies and review articles were examined. The study protocol was previously registered in PROSPERO (registration number CRD42023460205), and the meta-analysis was reported based on the PRISMA criteria.

### 2.2. Selection Criteria

Only studies that adhered to the following Population, Intervention, Comparison, and Outcome criteria were included in our research:

(1) Population: Studies must involve adult patients with HNC. There are no restrictions regarding the stage of the tumor at diagnosis, allowing for a more inclusive and comprehensive analysis.
(2) Intervention: The intervention of interest is aimed at assessing the impact of low GNRI on patient outcomes. The included studies should have evaluated the GNRI before the initiation of any type of anticancer therapy, such as surgical interventions or radiotherapy. This factor is crucial to obtaining an unaltered baseline that is not influenced by the effects or side effects of cancer treatments.
(3) Comparison: Patients with normal/high GNRI served as the control group.
(4) Outcome: The primary outcome of interest is overall survival. To be included, studies should either directly report HRs along with their 95% confidence intervals (CIs) for overall survival or provide sufficient data to allow for the calculation of these statistics.

Conversely, studies that met the following exclusion criteria were omitted:

(1) Nature of Publication: Letters, editorials, reviews, case studies, or conference abstracts are excluded as these might not provide the comprehensive data required for our meta-analysis.
(2) Insufficient Data: Any study that failed to provide the data to determine the correlation between pretreatment GNRI and overall survival is excluded.
(3) Focus on Esophageal Cancer: Our research is explicitly concentrated on HNC. As a result, any study mainly targeting esophageal cancer is not considered.

### 2.3. Data Extraction

Using a standardized data collection form, two coauthors independently performed data extraction. The collected data included the study details (e.g., the country where the study was conducted, first author, year of publication, and sample size), pathological attributes (e.g., tumor–node–metastasis (TNM) staging], demographic characteristics of patients (e.g., age and sex), and clinical variables (e.g., survival outcomes, GNRI cutoff values, treatment modalities used, and follow-up period duration). For studies that provided both adjusted and non-adjusted HRs, we collected adjusted HRs for analysis. In cases where disagreements arose between the two authors, a third author served as the arbitrator to resolve the differences.

### 2.4. Outcomes and Definition

The primary outcome of this meta-analysis was the association between the GNRI and the overall survival rate. To assess overall survival, the GNRI was categorized into two groups: low and high. If the original studies divided the GNRI into three or four categories, we selected the lowest and highest values for comparison in our analysis. As regards secondary outcomes, we assessed the correlation between the overall survival rate and other predictors, focusing on variables for which more than four studies provided sufficient details to calculate the associated risk. In this meta-analysis, the selected variables were age, male sex, tumor stage, T-stage, and *N*-stage.

### 2.5. Quality Assessment

Two reviewers independently evaluated the methodological integrity of the selected studies using the Newcastle–Ottawa Scale (NOS) designed for cohort studies. This assessment tool focuses on three key areas of the selection process for study groups, the comparability between these groups, and the manner wherein outcomes are assessed. Studies that received a score of seven or higher were considered to be of high quality. Disagreements were resolved through discussion and agreement.

### 2.6. Statistical Analysis

The relationship between pretreatment GNRI and overall survival was expressed as pooled HRs along with their 95% CIs using a random-effects model. This model was selected to accommodate the potential variability or heterogeneity existing among the included studies, providing a more generalized inference. The degree of heterogeneity among the included studies was measured using the *I*^2^ statistic. This metric is pivotal in the evaluation of the percentage of total variation across studies due to heterogeneity rather than chance. An *I*^2^ value exceeding 50% was interpreted as indicative of substantial heterogeneity, prompting a more meticulous examination of the individual study characteristics and potential sources of variability. Contrarily, an *I*^2^ value below 25% indicated minimal heterogeneity, suggesting that the variations among study estimates were predominantly caused by sampling error. Visual examination of funnel plots was employed to assess potential publication bias when more than ten studies were available for a single outcome. In general, funnel plots serve as graphical tools for identifying asymmetry, which can be indicative of publication bias, small-study effects, or both. The presence of publication bias suggests that studies with negative findings are less likely to be published, potentially skewing the overall results of the meta-analysis. Sensitivity analyses, excluding one study at a time, were conducted to assess the robustness of our findings. This method enables evaluation of the influence of each individual study on the overall pooled estimate, determining whether the exclusion of any single study would lead to a substantial alteration in the results. By conducting these analyses, we aimed to assess the robustness of our findings, ensuring that our conclusions are not disproportionately driven by any particular study included in the meta-analysis. To assess the association between covariates (e.g., sample size, male proportion, and follow-up time) and effect sizes as previously reported [23,24], meta-regression was conducted. These particular covariates were chosen as they frequently exhibit significant variation across studies. Such a variation could, in turn, contribute to and account for the heterogeneity in the combined results. All meta-analyses were conducted using RevMan and Comprehensive Meta-Analysis using V3 (BioSTAT, Englewood, NJ, USA). A result of *p* < 0.05 was considered to indicate statistical significance.

## 3. Results

### 3.1. Study Screening and Characteristics of Studies

**Figure 1:**
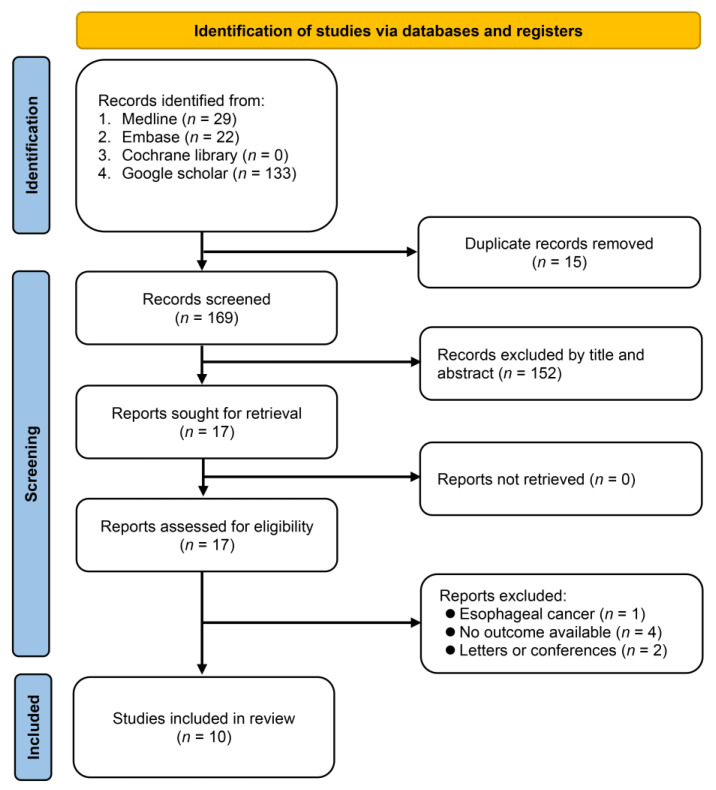
Flowchart of the study selection.

Figure 1 presents the study screening in the current meta-analysis. The initial database query yielded 184 articles. After eliminating duplicates (*n* = 15), 169 articles were left for further examination. Two separate reviewers scrutinized the titles and abstracts of the articles and excluded 152 due to the lack of relevance to the research topic, leaving 17 articles for full-text review. Of these, seven were subsequently eliminated, mainly because they either did not report pertinent outcomes (only addressing perioperative complications without survival analysis) or were published as conference abstracts. Finally, ten retrospective studies [20,21,22,25,26,27,28,29,30,31] fulfilled the eligibility requirements and were incorporated into the final qualitative and quantitative analyses.

The characteristics of the studies included in the analysis are summarized in Table 2. All studies were published between 2021 and 2023, reflecting recent interest in this research area. Eight studies provided information on mean or median age, with a range of 45 to 72.1 years [20,21,22,25,26,27,30,31]. Two studies reported that the proportions of patients aged over 70 and 65 years were 61.3% and 87.2%, respectively [28,29]. The studies predominantly featured male patients (proportion of male sex, 61.3%–91%), reflecting higher HNC incidence among them. The total number of patients across the studies was 2793, with most studies (*n* = 8) including more than 100 patients (sample size range, 61–1065 participants) [20,22,25,26,28,29,30,31]. The tumor stage of the patients varied across studies: five studies included patients with tumor stages I–IV [20,26,27,28,29], two focused on tumor stages III–IV [25,30], and one study included patients with tumor stages II–III [31]. Two studies did not specify the tumor stage of the patients [21,22]. Furthermore, six provided adjusted HRs that controlled for potential confounders, such as age, sex, and tumor stage, in their multivariate analyses [20,21,25,28,29,31], whereas the remaining studies reported only unadjusted HRs [22,26,27,30]. The GNRI cutoff values varied, ranging from 82 to 107.7, with several studies using common values, including 92 or 98, for reference. Follow-up durations were extended from 12 months to 5 years, and the majority of the studies were conducted in Asian countries, particularly Japan (*n* = 6), China (*n* = 2), and Taiwan (*n* = 1).

Table 2 summarizes the risk of bias across all studies assessed using the NOS. Overall, the NOS scores varied from 5 to 8. Four of the studies were categorized as low quality, with scores ranging from 5 to 6, indicating a higher risk of bias [21,27,28,30].

### 3.2. Outcomes

#### 3.2.1. Primary Outcomes

**Figure 2:**
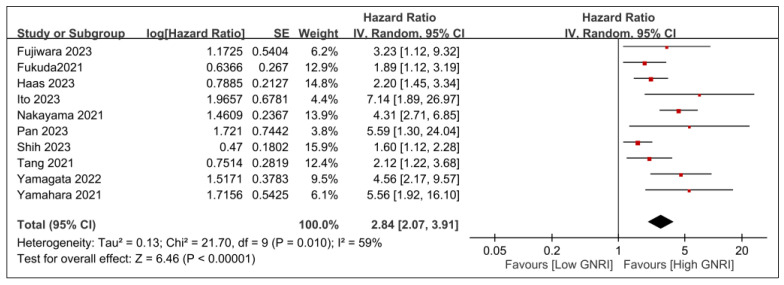
Forest plot showing the association between the geriatric nutritional risk index (GNRI) and overall survival: IV, inverse variance; CI, confidence interval; SE, standard error [20,21,22,25,26,27,28,29,30,31]. Black diamonds: Overall effect size and its confidence interval. Red squares: Individual study effect sizes. The size of the square represents the weight of the study in the meta-analysis.

**Figure 3:**
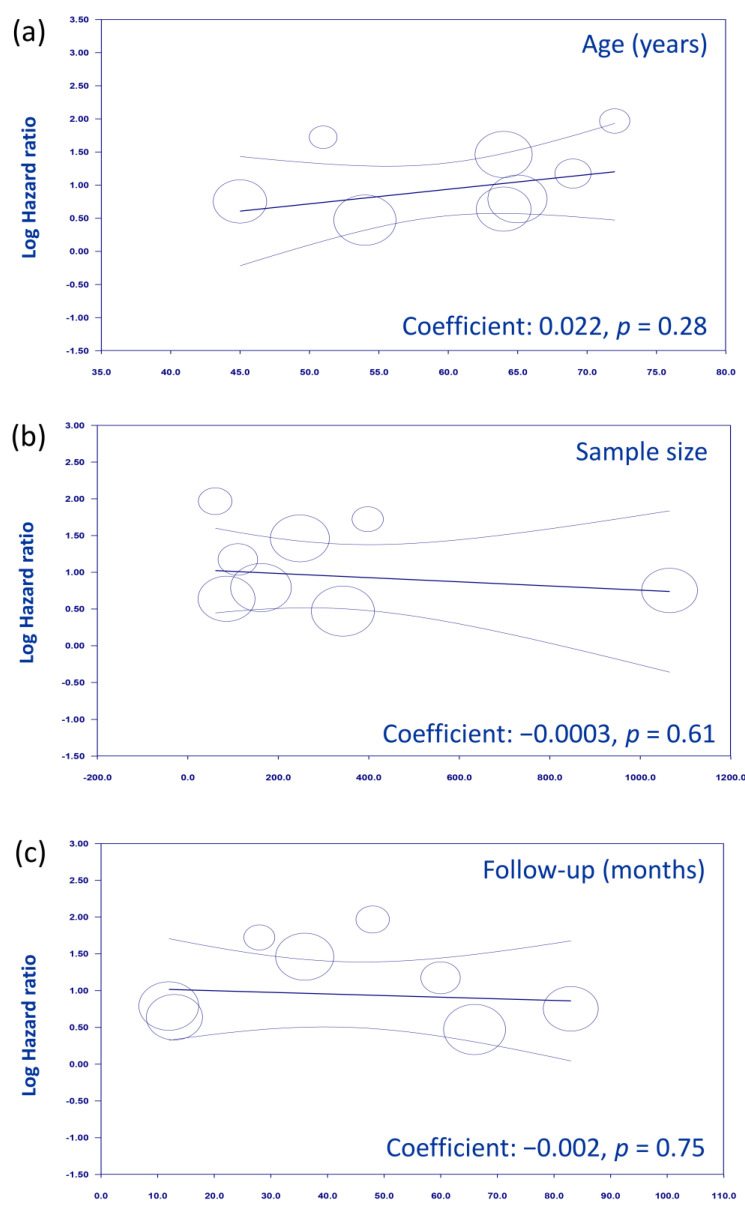
A meta-regression analysis exploring the relationship between overall survival and various covariates, including age (**a**), sample size (**b**), and follow-up duration (**c**).

**Figure 4:**
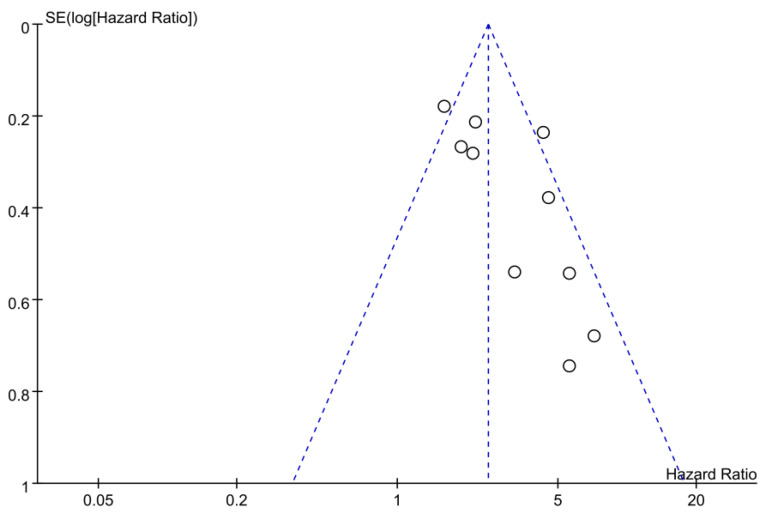
Funnel plot illustrating the potential for publication bias.

The meta-analysis of 10 studies involving 2793 patients revealed that low GNRI was associated with a significantly worse survival rate than high GNRI, with an HR of 2.84 (95% CI, 2.07–3.91; *p* < 0.00001) and moderate heterogeneity (*I*^2^ = 59%) (Figure 2). Sensitivity analysis, conducted by excluding one study at a time, upheld the consistency of these results. We further assessed the associations between other covariates (e.g., age, sample size, and follow-up duration) and the effect size. There was no correlation between the effect size and these covariates, including age (*p* = 0.28) (Figure 3a), sample size (*p* = 0.61) (Figure 3b), and follow-up duration (*p* = 0.75) (Figure 3c). A potential risk of publication bias was demonstrated by an asymmetric funnel plot (Figure 4).

**Figure 5:**
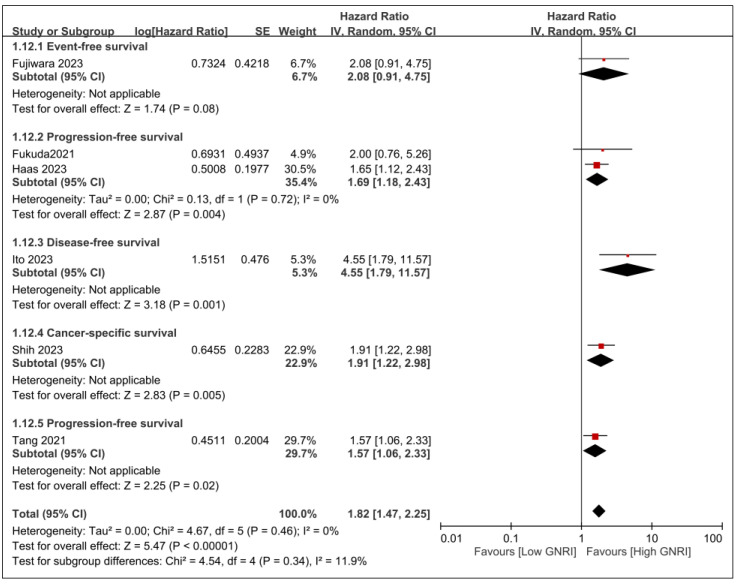
Forest plot illustrating the association between the GNRI and cancer-related diverse survival outcomes [20,21,22,25,27,31]. Black diamonds: Overall effect size and its confidence interval. Red squares: Individual study effect sizes. The size of the square represents the weight of the study in the meta-analysis.

Other cancer-related survival outcomes are presented in Figure 5. Overall, patients with low GNRI were at risk for worse cancer-related survival outcomes than those with high GNRI (HR, 1.82; 95% CI, 1.47–2.25; *p* < 0.00001; *I*^2^ = 0%) (Figure 5).

#### 3.2.2. Secondary Outcomes

**Figure 6:**
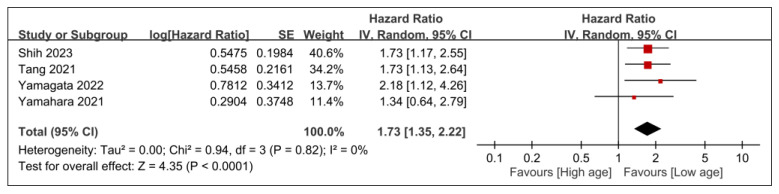
Forest plot showing the association between age and overall survival. IV, inverse variance; CI, confidence interval; SE, standard error [25,28,29,31]. Black diamonds: Overall effect size and its confidence interval. Red squares: Individual study effect sizes. The size of the square represents the weight of the study in the meta-analysis.

**Figure 7:**
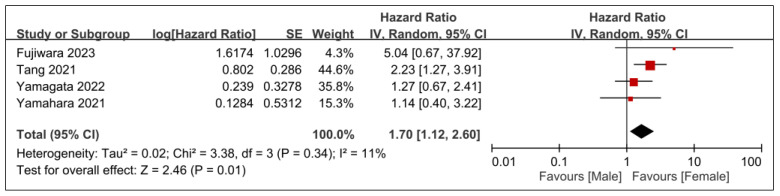
Forest plot showing the association between male sex and overall survival. IV, inverse variance; CI, confidence interval; SE, standard error [20,28,29,31]. Black diamonds: Overall effect size and its confidence interval. Red squares: Individual study effect sizes. The size of the square represents the weight of the study in the meta-analysis.

The associations between overall survival and patient-related factors, including age and sex, are presented in Figure 6 and Figure 7. Older patients were more likely to have worse overall survival than their younger counterparts (HR, 1.73; 95% CI, 1.35–2.22; *p* < 0.0001; *I*^2^ = 0%) (Figure 6). Furthermore, male sex was identified as a risk factor for diminished overall survival (HR, 1.7; 95% CI, 1.12–2.6; *p* = 0.01; *I*^2^ = 11%) (Figure 7).

**Figure 8:**
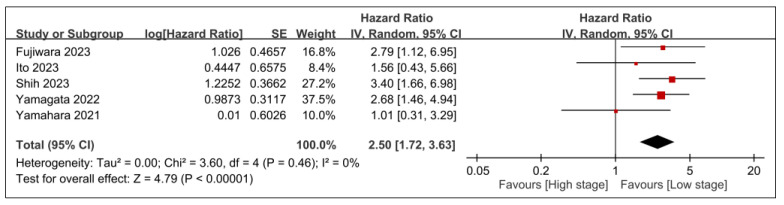
Forest plot showing the association between tumor stage and overall survival. IV, inverse variance; CI, confidence interval; SE, standard error [20,25,27,28,29]. Black diamonds: Overall effect size and its confidence interval. Red squares: Individual study effect sizes. The size of the square represents the weight of the study in the meta-analysis.

**Figure 9:**
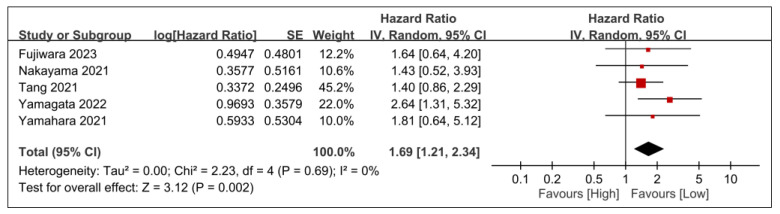
Forest plot showing the association between T-stage and overall survival. IV, inverse variance; CI, confidence interval; SE, standard error [20,28,29,30,31]. Black diamonds: Overall effect size and its confidence interval. Red squares: Individual study effect sizes. The size of the square represents the weight of the study in the meta-analysis.

**Figure 10:**
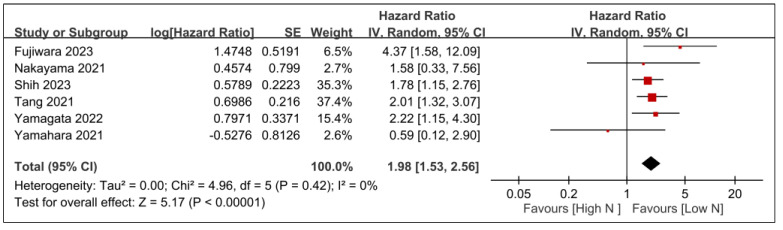
Forest plot showing the association between *N*-stage and overall survival. IV, inverse variance; CI, confidence interval; SE, standard error [20,25,28,29,30,31]. Black diamonds: Overall effect size and its confidence interval. Red squares: Individual study effect sizes. The size of the square represents the weight of the study in the meta-analysis.

The relationships between overall survival and other tumor-related prognostic factors, such as tumor stage, T-stage, and *N*-stage, are demonstrated in Figure 8, Figure 9 and Figure 10. Advanced tumor stage was significantly linked to poorer survival rates (HR, 2.5; 95% CI, 1.72–3.63; *p* < 0.00001; *I*^2^ = 0%) (Figure 8). A sensitivity analysis corroborated this consistent result. Moreover, patients with elevated T-stage (HR, 1.69; 95% CI, 1.21–2.34; *p* = 0.002; *I*^2^ = 0%) (Figure 9) or *N*-stage (HR, 1.98; 95% CI, 1.53–2.56; *p* < 0.00001; *I*^2^ = 0%) (Figure 10) exhibited worse overall survival than those in lower categories. The findings were further validated through a sensitivity analysis, confirming the consistency of the correlation between higher tumor stages (e.g., T- or *N*-stage) and poorer overall survival.

In light of the calculated HRs, advanced tumor stage was identified as a more potent predictor of adverse outcomes than other risk factors, which exhibited HRs ranging from 1.69 to 1.98.

## 4. Discussion

This meta-analysis, conducted on studies published between 2021 and 2023, examined 2793 patients to explore the association between lower GNRI and prognostic outcomes in individuals with HNC. Compared with a higher GNRI, a lower GNRI was significantly correlated with reduced overall survival, as evidenced by a 2.84 HR. Meta-regression analyses did not show a significant correlation between the observed effect size and selected covariates, including age, sample size, and follow-up duration. Age emerged as a significant prognostic factor, with older patients demonstrating poorer overall survival than younger ones. Furthermore, male patients were at a greater risk for poor overall survival. Also, compared with those categorized at lower stages, patients with advanced tumor stages and elevated T- or *N*-stages were statistically associated with unfavorable survival outcomes.

Malnutrition and muscular deficits are prognostic factors in HNC that impact clinical outcomes and, potentially, immune function. Malnutrition, assessed using tools such as the Patient-Generated Subjective Global Assessment, is prevalent in 30–50% of patients with HNC and is associated with advanced tumor stages and decreased survival rates [32,33]. Sarcopenia, alternatively, affects 7–71% of patients with HNC and is associated with decreased overall survival, increased toxicity from chemoradiotherapy, and higher hospital admission rates [34]. Poor nutritional status is strongly associated with worse prognosis in patients with cancer for several reasons. First, malnutrition in these patients causes nutrient deficiencies that weaken the immune system, thereby hindering the body’s ability to target and eliminate cancer cells, negatively affecting prognosis [35,36,37]. Second, malnutrition exacerbates inflammation and, when accompanied by metabolic changes inherent to cancer, leads to a proinflammatory state characterized by elevated cytokine levels, further driving cancer progression [38,39]. Last, malnutrition hampers treatment tolerance; patients with malnutrition frequently have lower physiological reserves and compromised organ function, reducing their ability to endure essential but harsh treatments, such as chemotherapy and radiation [40].

In the current meta-analysis, a lower GNRI was significantly correlated with inferior overall survival outcomes than a higher GNRI. A low GNRI could indicate compromised nutritional status, heightened inflammatory responses, and diminished antitumor immunity. These factors may collectively contribute to an unfavorable prognosis in patients with HNC. Our results highlight the detrimental effects of malnutrition on cancer-related outcomes, corroborating previous meta-analytical evidence indicating that a low pretreatment prognostic nutritional index (PNI) is significantly associated with reduced overall survival [41]. The meta-analysis that synthesized data from 14 studies involving 7815 patients revealed that a low pretreatment PNI was close to a twofold higher risk of mortality (e.g., an HR of 1.93) [41]. In the current meta-analysis, the pooled HR was 2.84, indicating close to a threefold higher risk of mortality for patients with a low GNRI compared with those with a high GNRI. The GNRI may serve as a more robust predictor of overall survival than the PNI. The ability to risk-stratify patients using this simple, inexpensive, and routinely available index could guide treatment decisions and follow-up care. For example, higher-risk patients identified by their low GNRI scores may benefit from aggressive nutrition intervention or intensive monitoring during treatment. Although our results were predominantly obtained from studies conducted in Asian countries, their applicability to other ethnic populations may be feasible, particularly considering the universal association between malnutrition and cancer outcomes.

In the current meta-analysis, older age, male sex, advanced tumor stages, and elevated T- or *N*-stages were identified as significant prognostic factors associated with poorer overall survival. Based on the observed HRs, advanced tumor stage was identified as a more potent prognostic indicator (HR, 2.5) than the other assessed risk factors, which exhibited a range of HRs between 1.69 and 1.98. Patients with a low GNRI are at an approximately threefold higher risk of mortality (HR, 2.84), indicating that the GNRI could be a critical factor for assessing survival prognosis, possibly even more potent than other tumor- or patient-specific variables. The TNM staging system is widely used in HNC for prognostics and treatment planning. However, its focus on anatomical tumor characteristics indicates that it may not completely capture other factors influencing survival. Consequently, patients with the same TNM stage can experience varying outcomes, indicating the need for a more comprehensive prognostic approach. Based on the findings of our meta-analysis, a low GNRI could also be considered a critical factor in risk stratification and patient management for HNC beyond the TNM staging system.

Aside from the GNRI and PNI, several malnutrition/inflammation markers for prognosis prediction in patients with HNC have been reported. A recent meta-analysis that incorporated nine studies involving 3211 patients with HNC revealed a twofold increase in mortality among patients who had low pretreatment albumin-to-globulin ratios (HR, 2.18) [42]. Another meta-analysis involving 4881 patients with HNC revealed that higher pretreatment neutrophil-to-lymphocyte ratios were associated with a 1.5-fold increase in mortality risk [17]. A meta-analysis that included six studies involving 2169 patients with nasopharyngeal carcinoma revealed that a high systemic immune-inflammation index was significantly associated with an approximately 1.7-fold increase in mortality rate (HR, 1.69) [43]. In a meta-analysis of seven selected studies involving 1059 patients undergoing radiotherapy for HNC, pretreatment sarcopenia was associated with a twofold increase in mortality rate (HR, 2.07) [44]. Based on a review of the existing literature and the findings of the current meta-analysis, the GNRI appeared to be a promising prognostic marker for patients with HNC.

Current evidence suggests that the GNRI can be used to predict overall survival in various cancer types. In a meta-analysis of 11 studies involving 1785 patients with esophageal cancer, the pooled results indicated that a lower GNRI adversely affected overall survival with an HR of 1.75 [45]. Furthermore, in a meta-analysis of 11 studies involving 2865 patients with non-small cell lung cancer, a low GNRI was found to be a significant predictor of poor overall survival with an HR of 1.96 [19]. By incorporating data from six studies, another meta-analysis showed that a low GNRI is significantly correlated with reduced overall survival in pancreatic cancer, with a pooled HR of 1.95 [46]. Furthermore, a meta-analysis of eight retrospective studies involving 3239 patients with colorectal cancer revealed that malnutrition, defined by a GNRI cutoff of 98, was associated with decreased overall survival, with an HR of 1.66 [18]. These findings suggest that a low GNRI is typically correlated with a doubled mortality rate in various cancers. Contrarily, our results showed a threefold increase in mortality rate in patients with HNC having a low GNRI, suggesting that using the GNRI to predict HNC prognosis is more effective than for other cancers.

To our knowledge, this is the first meta-analysis that specifically investigated the association between the GNRI and outcomes in patients with HNC. One strength of the current meta-analysis is that the majority of the included studies involved Asian populations, thereby minimizing the potential confounding effects of ethnic variability. In addition, the relatively low heterogeneity level among the included studies strengthens the validity of our conclusions. Furthermore, to investigate the relationship between the effect size and various covariates, including sample size, age, and follow-up duration, we used meta-regression techniques. The absence of any significant correlations strengthens the validity of our conclusions.

Despite the promising implications of the GNRI as a predictive tool for prognosis, acknowledging the limitations inherent in our meta-analysis is essential. First, the pooled analysis combining both adjusted and unadjusted HRs is a limitation, as the unadjusted results may be subject to confounding. Moreover, while adjusted HRs were used for analyses, the variables incorporated in these analyses of HRs differed across studies, potentially affecting the reliability of the results. Second, the majority of studies focused on Asian cohorts, thereby possibly restricting the generalizability of our findings to other ethnic or racial groups. Third, the optimal GNRI cutoff value for prognosis prediction remained unknown, thereby limiting the broad applicability of our results. Fourth, all the included studies used retrospective designs, introducing potential bias into the findings. Furthermore, the key weakness of the retrospective data is the lack of direct, in-person verification of clinical details and disease progression for each patient. Prospective studies allowing for face-to-face patient evaluation over time would strengthen the validity of the results. Finally, there are potential risks of publication bias in the included studies. In light of these limitations, future research is warranted to validate our findings and establish an optimal GNRI cutoff value for survival outcome prediction in patients with HNC.

## 5. Conclusions

In summary, our findings highlight that a low pretreatment GNRI is significantly associated with poor overall survival in patients with HNC, resulting in an approximately threefold increase in mortality risk. Considering its ease of measurement, the GNRI could serve as a cost-effective biomarker for both prognostic assessment and individualized treatment planning. To substantiate these findings and assess the clinical utility of the GNRI, future research should include additional randomized multicenter prospective trials and explore the potential benefits of nutritional supplementation based on the GNRI values to improve clinical outcomes.

## Figures and Tables

**Table 1:** Search strategy for Medline.

1	((“gingival” or “Lip” or “Palatal” or “Salivary Gland” or “Tongue” or “Otorhinolaryngologic” or “Ear” or “Laryngeal” or “Nose” or “Pharyngeal” or “Parathyroid” or “Squamous Cell Carcinoma of Head and Neck” or “Thyroid” or “Thyroid” or “Tracheal” or “oral” or “larynx” or “pharynx”) adj3 (Neoplasm or tumor or cancer)).mp.
2	exp “Head and Neck Neoplasms”/
3	(“Geriatric Nutritional Risk Index” or GNRI).mp.
4	(“Overall survival” or “Prognosis” or “Mortality” or “Disease-Free Survival” or “Progression-Free Survival”).mp.
5	exp “Survival”/or exp “Mortality”/or exp “Disease-Free Survival”/or exp “Progression-Free Survival”/
6	(1 or 2) and 3 and (4 or 5)

**Table 2:** Characteristics of the included studies (*n* = 10).

	Age (Years)	Male (%)	*n*	Tumor Stage	Treatment	GNRI Cutoff Values	Follow-Up	Country	NOS
Fujiwara 2023 [20]	69 (67–71)	79	111	I–IV	CRT	98	5 yrs	Japan	7
Fukuda 2021 [21]	64 (32–77)	87.2	86	NA	Platinum + fluorouracil + cetuximab	98	13.2 m	Japan	5
Haas 2023 [22]	65 (28–85)	71	162	NA	Immune checkpoint inhibitors	92	12 m	Austria	8
Ito 2023 [27]	72.1 ± 5.4	70.5	61	I–IV	Radical surgery	93.7	48.3 m	Japan	6
Nakayama 2021 [30]	64 (29–85)	85.9	248	III–IV	RT/surgery/CRT	92 vs. 98	36 m	Japan	5
Pan 2023 [26]	50.9 (44.5–57.0)	73.1	398	I–IV	Radiotherapy	82 vs. 98	2.3 yrs	China	8
Shih 2023 [25]	54 (30–59)	91	343	III–IV	Radical surgery	97.8	66.5 m	Taiwan	7
Tang 2021 [31]	45 (38–52)	72.40	1065	II–III	IMRT/CRT	107.7	83 m	China	7
Yamagata 2022 [28]	49.7% (>70 years)	61.30	155	I–IV	NA	98	36 m	Japan	6
Yamahara 2021 [29]	76.2% (>65 years)	87.20	164	I–IV	RT/surgery	82 vs. 98	53	Japan	7

GNRI, geriatric nutritional risk index; NOS, Newcastle–Ottawa Scale; NA, not available; RT, radiotherapy; IMRT, Intensity-Modulated Radiation Therapy; CRT, chemoradiation therapy; m, month; yrs, years.

## Data Availability

The original contributions presented in this study are included in this article; further inquiries can be directed to the corresponding authors.
